# Physical Characteristics and Physical Fitness Profiles of Korean Taekwondo Athletes: A Systematic Review

**DOI:** 10.3390/ijerph18189624

**Published:** 2021-09-13

**Authors:** Jeong-Weon Kim, Sang-Seok Nam

**Affiliations:** 1 Graduate School of Professional Therapy, Gachon University, 1342 Seongnam-daero, Sujeong-gu, Seongnam-si 13120, Korea; zeezone@gachon.ac.kr; 2 Taekwondo Research Institute, Kukkiwon, 32 Teheran 7-gil, Gangnam-gu, Seoul 06130, Korea

**Keywords:** taekwondo, physical characteristics, physical fitness, systematic review, normal distribution

## Abstract

This study aimed to present a standard and normal distribution of Taekwondo athletes’ physical characteristics and physical fitness profiles using a systematic review. A systematic search was conducted using four Korean databases (Research Information Sharing Service, National Digital Science Library, DBpia, and Korean Studies Information Service System). From 2010 to 2020, we reviewed 838 papers on Taekwondo athletes’ physical characteristics and physical fitness factors (e.g., body composition, muscle strength, muscular endurance, flexibility, cardiorespiratory fitness, power, agility, balance, speed, and reaction time). Of them, 24 papers were selected and analyzed. The criteria for selecting the physical characteristics and physical fitness factors for data extraction were set to have a total sample size of more than 30 individuals and included two or more studies. The sample size and average and standard deviation of physical characteristics and physical fitness factors were extracted from each selected study. In this study, the estimation error of all variables, except for the eyes-closed single-leg stance (15.71%), was less than 8%. Therefore, it was confirmed that there was no problem with the validity of the estimated values. These results could be used as an essential objective basis for evaluating the physical characteristics and physical fitness profiles of Taekwondo athletes in most countries worldwide and setting training goals.

## 1. Introduction

Taekwondo is an international martial arts sport conducted in 210 countries worldwide as an official Olympic sport. A Taekwondo competition occurs in three rounds, with a duration of 2 min per round and a rest duration of 1 min between rounds [1]. Athletes who score more points or knock out their opponent win. During a competition, athletes use powerful and fast kicks and punches on their opponent’s trunk and sometimes kicks to the face [2]. These movements are high-intensity anaerobic or aerobic exercises that induce powerful lower-extremity movements [3]. In addition, agility, flexibility, and muscular endurance are required to maintain an excellent performance among Taekwondo athletes [4,5,6]. Therefore, it is necessary to manage physical fitness factors to improve their performance [7]. This can be achieved by accurately evaluating the level of fitness of athletes and setting goals. Athletes need to know their physical characteristics and physical fitness profiles for effective training because high levels of physical fitness can affect their exercise performance [8]. Suppose there is a basis for the standard distribution of physical fitness profiles necessary for the characteristics of sports events. In this case, it can be used to evaluate athletes’ fitness levels and set training goals. Although the physical fitness profiles of taekwondo athletes have been well described in the previous studies, no studies have examined the standard distribution of physical fitness [9,10,11].

Heller et al. [9] compared the physical fitness factors of 23 national Taekwondo athletes from the Czech Republic to those of the general public. Meanwhile, Marković [10] divided 13 women from the Croatian national Taekwondo team into medal-winning and non-medal-winning athletes at World Championships or Olympic Games, comparing the physical fitness profiles between them. In addition, Mathunjwa et al. [11] studied the physical characteristics of 36 internationally ranked junior Taekwondo athletes; the physical fitness test results were standardized in z-scores, which were then compared among the athletes. Furthermore, Bridge et al. [12] and da Silva Santos et al. [13] reported the physical characteristics and physical fitness of Taekwondo athletes using a systematic review but did not present any quantitative results.

Previous studies have provided information on the physical characteristics and physical fitness of Taekwondo athletes [12,13]. However, it is difficult to use them as a specific indicator because there is no standard distribution to evaluate the level of physical fitness of Taekwondo athletes. Standard distribution data are needed to determine the mean and percentile values of Taekwondo athletes’ physical characteristics and physical fitness parameters. In general, a standard distribution is meaningful when the measurement results of a large sample form a normal distribution [14]. However, it can be analyzed via a systematic review using the measured variables in a previous study [15]. In other words, the results could be interpreted as a normal distribution when the sum of sample sizes is sufficiently large by integrating each previous study [15]. Nevertheless, the validity and reliability of the resulting values can be questioned if different prior studies have different measurement tools. However, the measurement of physical characteristics and physical fitness variables has become common worldwide. Furthermore, the systematic review method can resolve the concerns about reliability and validity by eliminating extreme values when integrating the results of variables [15]. Thus, the standard distribution of physical characteristics and physical fitness factors can be estimated using the pooled mean and pooled standard deviation from previous studies.

The purpose of this study was to present a standard and normal distribution of Taekwondo athletes’ physical characteristics and physical fitness profiles using a systematic review.

## 2. Materials and Methods

### 2.1. Search Strategy

A systematic search was conducted using four Korean databases. Korean taekwondo athletes were selected as the research subjects because they have the best performance in the world. We searched the Research Information Sharing Service (RISS), National Digital Science Library (NDSL), DBpia, and Korean Studies Information Service System (KISS), using the following terms: “Taekwon”, “athlete”, “physical fitness”, and Korean terms. Data were collected up to December 2020 and regularly updated manually. At the initial stage of screening, articles published before 31 December 2009 were excluded. The study was conducted in accordance with the guidelines of the Declaration of Helsinki and was approved by the Institutional Review Board of Konkuk University (7001355-201804-E-077).

### 2.2. Inclusion and Exclusion Criteria

In the first step we input the database search results to Microsoft Excel 2019 and performed duplicate elimination. This study focused on describing the physical characteristics and physical fitness profiles of Korean Taekwondo athletes. For this reason, all papers on the physical characteristics and physical fitness of Korean Taekwondo athletes were included in this study. Articles were excluded for the following reasons: (1) data aside from physical characteristics; (2) data aside from physical fitness; (3) irrelevant data for analysis; (4) non-competition Taekwondo athletes; (5) unavailable full-text; (6) duplicates; and (7) conference presentations, case reports, commentaries, and review articles. Two independent authors evaluated the eligibility of each item. In addition, qualified articles were collected for data extraction steps via reading and evaluation of the full text of each paper. Finally, an article suitable for the data extraction process was included.

### 2.3. Data Extraction

Two independent authors performed the data extraction. The criteria for selecting the physical characteristics and physical fitness factors for data extraction were set to have a total sample size of more than 30 individuals and included two or more studies. The sample size and average and standard deviation of the physical characteristics and physical fitness factors were extracted from each selected study.

### 2.4. Data Calculation and Statistical Analysis

#### 2.4.1. Pooled Mean Calculation

Two independent authors performed the data extraction. The pooled mean was calculated using the mean and sample size from the final selected study using Formula (1).

(1) $${\mathrm{Mean}}_{\mathrm{pooled}}=\frac{\left(m_{1}\times n_{1}\right)+\left(m_{2}\times n_{2}\right)+\left(m_{3}\times n_{3}\right)+\ldots +\left(m_{i}\times n_{i}\right)}{n_{1}+n_{2}+n_{3}+\ldots +n_{i}}$$

Formula (1). Calculation formula for the pooled mean. Note. m: mean of each study; n: sample size of each study; and i: number of studies.

#### 2.4.2. Pooled Standard Deviation Calculation

The pooled standard deviation was calculated using the standard deviation and sample size from the final selected study using Formula (2).

(2) $${\mathrm{SD}}_{\mathrm{pooled}}=\sqrt{\frac{\left(n_{1}-1\right)\times {S_{1}}^{2}+\left(n_{2}-1\right)\times {S_{2}}^{2}+\ldots +\left(n_{i}-1\right)\times {S_{i}}^{2}}{n_{1}+n_{2}+\ldots +n_{i}-i}}$$

Formula (2). Calculation formula for the pooled standard deviation. Note. SD: standard deviation; n: sample size of each study; S: standard deviation of each study; and i: number of studies.

#### 2.4.3. Estimated Physical Characteristic and Physical Fitness Value Calculation

The estimated values were calculated using the pooled mean, pooled standard deviation, and Z-score in the cumulative normal distribution (Table 1) using Formula (3).

(3) $$\mathrm{Estimated}\;{\mathrm{Value}}_{p\%}={\mathrm{Mean}}_{\mathrm{pooled}}+Z_{p\%}\times {\mathrm{SD}}_{\mathrm{pooled}}$$

Formula (3). Calculation formula for the estimated values*_p_*_%_. Note. *p*%: cumulative probability in the normal distribution, Z*_p_*_%_: Z-score of cumulative *p*%.

#### 2.4.4. Estimation Error Calculation

The estimation error was calculated using the sample size, standard deviation, and Z-score for a confidence level of 95% using Formula (4).

(4) $${\mathrm{Error}}_{\mathrm{estimation}}=\pm 1.96\times \frac{\sigma }{\sqrt{n}}$$

Formula (4). Calculation formula for the estimation error. Note. n: sample size, σ: standard deviation.

## 3. Results

### 3.1. Study Selection

**Figure 1:**
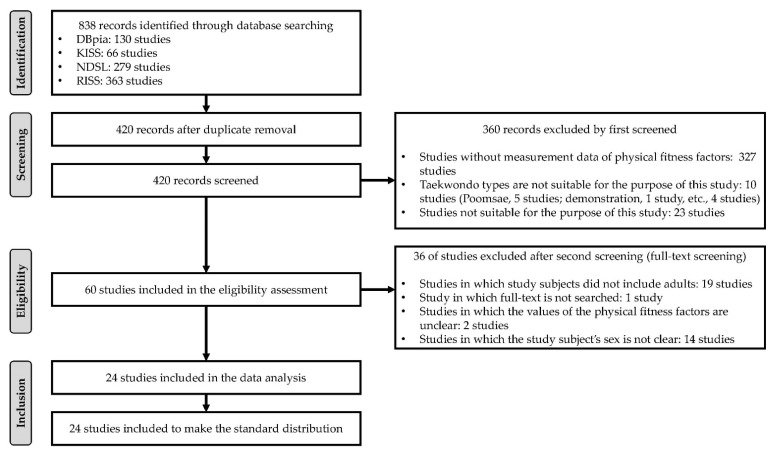
Flow diagram of the study screening and selection processes.

After a systematic search that selectively focused on clinical trials and cross-sectional studies, we retrieved 130, 66, 279, and 363 articles from DBpia, KISS, NDSL, and RISS, respectively. During the screening phase, 389 duplicate records and 29 conference presentations were excluded from the 838 articles. In addition, 327 not presenting physical characteristics and physical fitness data, 10 focusing on non-competition Taekwondo athletes, and 23 reporting irrelevant data for analysis were excluded from the 420 articles. Finally, 19 not including adults, 1 with unavailable full-text, 2 with unclear physical fitness data, and 14 with unclear patient sex were excluded from the 60 remaining articles. Twenty-four articles were then included after the screening and selection processes. The screening and selection processes are shown in Figure 1.

### 3.2. Study Characteristics

The study characteristics of the selected literature are listed in Table 2. Data were extracted from 22 studies on male Taekwondo athletes (*n* = 430, age: 20.10 ± 1.00 years, career: 5–15 years) and 7 studies on female Taekwondo athletes (*n* = 99, age: 19.40 ± 1.18 years, career: approximately 7.7 years). The variables involving a total sample size of more than 30 were extracted for each physical characteristic and physical fitness factor. As a result, 37 variables were extracted from studies on male Taekwondo athletes and 28 from studies on female Taekwondo athletes. We reviewed whether the final selected literature could allow an analysis of the physical characteristics and physical fitness factors by weight class; however, no weight class information was described. We attempted to classify the weight classes by the weight values from previous studies, although errors can occur because of the mean values. Therefore, weight classification was not analyzed, considering the validity of the resulting values. In addition, the side-step variables were excluded from the analysis owing to a lack of reliability because each study had different metrics. We then considered separating the excellent Taekwondo athletes from the non-excellent Taekwondo athletes. However, we failed to analyze them because the number of studies was very small.

### 3.3. Pooled Mean Value and Estimated Error

#### 3.3.1. Physical Characteristics

For the male Taekwondo athletes, the total sample size in relation to the physical characteristics was 224–430, and the estimated error was ±0.28–2.79%. The estimated error was the smallest height and the largest percentage of body fat. In addition, the estimated error of body mass index (BMI) (*n* = 224, ±0.89%) was smaller than that of the percentage of body fat (*n* = 236, ±2.79%).

For the female Taekwondo athletes, the total sample size in relation to the physical characteristics was 82–99, and the estimated error was ±0.64–3.05%. The estimated error was the smallest height and the largest percentage of body fat. The pooled and estimated error statistics for each variable are listed in Table 3.

#### 3.3.2. Physical Fitness Variables

For the male Taekwondo athletes, the total sample size in relation to the physical fitness variables was 42–203, and the estimated error was ±0.01–0.71%. The estimated error for all fitness variables was less than 8% without the eyes-closed single-leg stance (*n* = 65, ±15.71%). In addition, the estimated error of the maximal heart rate per minute (*n* = 67, ±1.26%) was smaller than that of the maximal oxygen consumption per minute (VO_2_max) (*n* = 129, ±2.49%).

The pooled and estimated error statistics of the physical fitness variables of the male Taekwondo athletes are shown in Table 4.

For the female Taekwondo athletes, the total sample size in relation to the physical fitness variables was 33–72, and the estimated error was ±1.94–7.44%. The estimated error for all fitness variables was less than 8%. The pooled and estimated error statistics of the physical fitness variables of the female Taekwondo athletes are shown in Table 5.

### 3.4. Estimated Normal Distribution and 95% Confidence Interval

#### 3.4.1. Physical Characteristics

The estimated values of each grade were calculated by applying the pooled mean and pooled standard deviation for each physical characteristic to the normal distribution and setting the grade at 10% intervals of cumulative probability. Examples of the estimated values corresponding to the top 10% of each physical characteristic in the study results were as follows: (1) the top 10% for the BMI of the male Taekwondo athletes was 20.0–20.4 kg/m^2^; and (2) the top 10% for the percentage of body fat of the female Taekwondo athletes was 18.3–19.8%. The estimated normal distribution and 95% confidence interval of each physical characteristic are listed in Table 6.

#### 3.4.2. Physical Fitness Variables

The estimated values of each grade were calculated by applying the pooled mean and pooled standard deviation for each physical fitness variable to the normal distribution and setting the grade at 10% intervals of cumulative probability.

Examples of the estimated values corresponding to the top 10% of each physical fitness variable in the study results were as follows: (1) the top 10% for the hand-grip strength of the male Taekwondo athletes was 49.1–51.5 kg; and (2) the top 10% for the VO_2_max of the female Taekwondo athletes was 61.2–63.8 mL/kg/min. The estimated normal distribution and 95% confidence interval of each physical fitness variable are listed in Table 7.

## 4. Discussion

For Taekwondo competitions, athletes must have excellent physical fitness, including aerobic capacity, anaerobic capacity, muscle strength, muscle endurance, flexibility, speed, and agility [9,10,39,40]. In addition, data-based exercise science information is helpful in improving Taekwondo athletes’ physical fitness and weakness [6]. Therefore, this study aimed to provide a profile of physical characteristics and physical fitness for Taekwondo competitors. To increase the value of this study’s data-based exercise science information, we secured the validity of the estimation results. In a previous study that developed an estimation model of the physical fitness level, the validity of the estimation results was recognized when the estimated error was within 8–10% [41,42,43]. In this study, the estimation error of all variables, except for the eyes-closed single-leg stance (15.71%), was less than 8%. Therefore, it was confirmed that there was no problem with the validity of the estimated values.

The following can be interpreted as the causes of the higher estimation error in the eyes-closed single-leg stance than in the other variables. First, the sample size in relation to the variable was small. The estimation error was calculated by dividing the standard deviation by the square root of the sample size; therefore, the smaller the sample size, the larger the estimation error. However, the total sample size for the eyes-closed single-leg stance was 65, so the sample size was not small compared to that of the other variables. Therefore, this problem is hardly attributable to the increase in the estimation error. Second, there was a large deviation between individuals in the measurement of the variables. The eyes-closed single-leg stance is a variable that shows a large individual difference in measurement. Therefore, the estimation error was calculated based on the eyes-closed single-leg stance data presented in a previous study.

Based on the results of the previous study, the estimated error of the eyes-closed single-leg stance was calculated to be 40.7% for 16 college soccer players (34.0 ± 28.21 s) [44] and 54.6% for 10 high school female volleyball players (59.5 ± 52.4 s) [45]. Therefore, the estimation error increases proportionally because the individual difference between the measurements is large in the eyes-closed single-leg stance test. However, the results of our study have general validity because the estimation error of all variables, except for the eyes-closed single-leg stance, was less than 8%.

The utilization of different measurements that evaluate the same physical fitness factors favoring indicators with small estimation errors may be preferred. However, they should be carefully selected considering the inherent reliability of the measurement methods. For example, selecting BMI should be avoided because it has a smaller estimation error than the percentage of body fat when measuring obesity. The percentage of body fat directly tested using the bioelectrical impedance method was more accurate in obesity assessment than BMI calculated based on height and weight [46,47]. Nevertheless, BMI is being used to assess obesity in the public health and sports fields. The results of this study may be fully utilized for evaluation because the error in the estimated BMI distribution was not significant. For sit-up tests, it is recommended to conduct such for 60 s with a lower estimation error than that for 30 s. Measurements via the same test method and reliability should utilize a distribution with a smaller estimation error. Nevertheless, sit-up tests for 30 s are also available in public health and sports because of the low estimation error.

Combat sports, such as Taekwondo, require high levels of physical fitness and physical characteristics [48]. Exercise program plans are important for improving and maintaining a high level of physical fitness suitable for the characteristics of Taekwondo events [49]. Taekwondo athletes should be conditioned to effectively manage and improve their physical fitness through systematic exercise programs [50]. Conditioning management requires detailed knowledge of the physiological and physical abilities required for competition [51,52]. Therefore, sports scientists and Taekwondo coaches should organize long-term and short-term training programs and provide objective feedback to motivate athletes. As in this study, objective collection and presentation of information on an athlete’s physical ability are important for feedback to the athlete [53]. The results of this study can help identify the physical profiles favorable to Taekwondo competitions and provide indicators of physical fitness standards for Taekwondo athletes [9,10]. This study had limitations. In the study, Korean Taekwondo athletes were considered the study subjects for the systematic search because they have the best performance in the world. However, Taekwondo athlete’s skills and performance are becoming similar around the world. Therefore, future studies need to analyze the physical characteristics and physical fitness factors of Taekwondo elite athletes worldwide.

## 5. Conclusions

This study estimated the standard distribution of each factor by aggregating previous studies measuring the physical characteristics and physical fitness variables of Taekwondo athletes in South Korea through a systematic literature review. This study found that almost all physical characteristics and the estimated distribution of the physical fitness variables were generally applicable (estimated error of less than 8%). These results could be an essential objective basis for evaluating Taekwondo athletes’ physical characteristics and physical fitness factors and setting training goals.

## Figures and Tables

**Table 1:** *Z*-score in the cumulative normal distribution.

*p*(%)	1%	5%	10%	20%	30%	40%	50%	60%	70%	80%	90%	95%	99%
*Z*	−2.33	−1.64	−1.28	−0.84	−0.52	−0.25	0.00	0.25	0.52	0.84	1.28	1.64	2.33

**Table 2:** Study characteristics and extracted outcome variables.

Sex	Study	Sample Size	Age (y)	Career (y)	Weight (kg)	Outcome Variables
Male	Cho et al., 2011 [16]	40	20.56 ± 1.25	>5	75.33 ± 8.68	Physiques (height, body fat); Cardiorespiratory endurance (VO_2_max, HRmax); Isokinetic muscular strength (left knee joint flexion at 60°/s, left knee joint extension at 60°/s, right knee joint flexion at 60°/s, right knee joint extension at 60°/s)
	Cho, 2020 [17]	67	19.69 ± 1.13	NR	70.48 ± 7.56	Physiques (height, BMI)
	Feng et al., 2020 [18]	27	19.7 ± 1.03	NR	70.5 ± 8.73	Physiques (height, BMI); Cardiorespiratory endurance (VO_2_max); Isokinetic muscular strength (peak anaerobic power)
	Hong et al., 2020 [19]	28	20.18 ± 10.8	9.61 ± 2.27	74.01 ± 8.29	Physiques (height, BMI, body fat, fat mass, LBM, thigh circumference); Muscular strength (back strength); Muscular endurance (sit-up per 60 s); Muscular power (standing long jump, vertical jump); Flexibility (backward flexion, sit and reach); Balance (eyes-closed single-leg stance); Agility (whole-body reaction time to light, whole-body reaction time to sound); Isokinetic muscular strength (average power, relative peak anaerobic power, absolute peak anaerobic, peak drop, total energy, left knee joint flexion at 60°/s, left knee joint extension at 60°/s, right knee joint flexion at 60°/s, right knee joint extension at 60°/s)
	Jang and Park, 2020 [20]	10	20.7 ± 0.95	NR	69.8 ± 1.17	Physiques (height, BMI, body fat); Muscular endurance (sit-up per 30 s, sit-up per 60 s); Muscular power (standing long jump); Flexibility (sit and reach); Isokinetic muscular strength (average power, relative peak anaerobic power, absolute peak anaerobic power, peak drop)
	Jang, 2020 [21]	10	20.7 ± 0.95	NR	69.8 ± 4.93	Physiques (height, BMI, body fat); Muscular endurance (sit-up per 30 s, sit-up per 60 s); Muscular power (standing long jump); Flexibility (sit and reach); Isokinetic muscular strength (average power, relative peak anaerobic power, absolute peak anaerobic power, peak drop)
	Jung, 2015 [22]	16	22.44 ± 0.96	NR	70.23 ± 6.28	Physiques (height); Muscular strength (back strength, hand grip); Muscular endurance (push-up per 60 s, sit-up per 60 s); Muscular power (vertical jump); Flexibility (sit and reach); Isokinetic muscular strength (left knee joint flexion at 60°/s, left knee joint extension at 60°/s, right knee joint flexion at 60°/s, right knee joint extension at 60°/s, left knee joint flexion at 180°/s, left knee joint extension at 180°/s, right knee joint flexion at 180°/s, right knee joint extension at 180°/s, left hip joint flexion at 60°/s, left hip joint extension at 60°/s, right hip joint flexion at 60°/s, right hip joint extension at 60°/s, left hip joint flexion at 180°/s, left hip joint extension at 180°/s, right hip joint flexion at 180°/s, right hip joint extension at 180°/s, trunk joint flexion at 60°/s, and trunk joint extension at 60°/s
Male	Kim and Lee, 2011 [23]	14	NR	≥10	71.01 ± 11.15	Physiques (height, BMI, body fat, LBM, and thigh circumference); muscular strength (back strength, left hand grip, and right hand grip); muscular power (vertical jump); flexibility (backward flexion, sit and reach); agility (whole-body reaction time to light, whole-body reaction time to sound); and isokinetic muscular strength (relative peak anaerobic power, peak drop)
	Kim et al., 2012 [24]	20	NR	NR	68.80 ± 8.29	Physiques (height, body fat); muscular strength (back strength); muscular endurance (sit-up per 30 s); muscular power (vertical jump); flexibility (sit and reach); and cardiorespiratory endurance (VO_2_max)
	Kwon and Cho, 2017 [25]	8	21.70 ± 1.50	≥10	66.50 ± 3.15	Physiques (height, body fat, and thigh circumference); muscular strength (hand grip); muscular endurance (sit-up per 60 s); muscular power (standing long jump, vertical jump); cardiorespiratory endurance (Harvard step test); flexibility (sit and reach); and balance (eyes-closed single-leg stance)
	Kwon and Cho, 2019 [26]	14	22.50 ± 1.56	10.45 ± 2.63	72.57 ± 2.20	Physiques (height); agility (eyes-closed single-leg stance); muscular strength (left hand grip, right hand grip); muscular endurance (sit-up per 30 s); muscular power (standing long jump, vertical jump); flexibility (sit and reach); and agility (whole-body reaction time to sound)
	Lee and Ham, 2018 [27]	13	20.00 ± 1.08	6.23 ± 1.23	72.57 ± 11.12	Physiques (height)
	Monks, 2016 [28]	17	19.78 ± 0.58	7.98 ± 2.13	74.46 ± 10.47	Physiques (height, body fat, and LBM); muscular endurance (sit-up per 60 s); muscular power (vertical jump); cardiorespiratory endurance (VO_2_max, HRmax, and all-out duration time); flexibility (sit and reach); and isokinetic muscular strength (average power, relative peak anaerobic power, absolute peak anaerobic power, peak drop, left knee joint flexion at 60°/s, left knee joint extension at 60°/s, right knee joint flexion at 60°/s, right knee joint extension at 60°/s, left knee joint flexion at 180°/s, left knee joint extension at 180°/s, right knee joint flexion at 180°/s, and right knee joint extension at 180°/s)
	Moon et al., 2016 [29]	11	19.64 ± 0.92	NR	74.29 ± 10.56	Physiques (height)
	Noh, 2015 [30]	31	19.60 ± 0.20	7.80 ± 0.30	72.50 ± 1.70	Physiques (height, BMI)
	Park & Yang, 2020 [31]	8	20.9 ± 1.06	NR	69.65 ± 2.35	Physiques (height, BMI, body fat); muscular endurance (sit-up per 30 s, sit-up per 60 s); muscular power (standing long jump); isokinetic muscular strength (average power, relative peak anaerobic power, absolute peak anaerobic power, and peak drop)
	Park, 2016 [32]	29	18.87 ± 0.24	NR	70.09 ± 4.11	Physiques (height, BMI, body fat, fat mass, and LBM); muscular strength (hand grip); muscular endurance (sit-up per 60 s); muscular power (standing long jump); cardiorespiratory endurance (20-m MST); flexibility (sit and reach); and agility (10-m two repeated run test)
	Seo et al., 2015 [7]	22	19.40 ± 0.95	9.50 ± 1.91	69.80 ± 9.46	Physiques (height, body fat, fat mass, and LBM); muscular power (standing long jump); cardiorespiratory endurance (20-m MST); flexibility (sit and reach); and isokinetic muscular strength (relative peak anaerobic power, and peak drop)
Male	Song et al., 2010 [33]	10	19.40 ± 1.20	NR	69.30 ± 3.80	Physiques (height, body fat, and fat mass); cardiorespiratory endurance (VO_2_max, HRmax); isokinetic muscular strength (relative peak anaerobic power, peak drop, left knee joint flexion at 60°/s, left knee joint extension at 60°/s, right knee joint flexion at 60°/s, right knee joint extension at 60°/s, left knee joint flexion at 180°/s, left knee joint extension at 180°/s, right knee joint flexion at 180°/s, and right knee joint extension at 180°/s)
	Tak et al., 2019 [34]	15	19.20 ± 0.78	>6	74.87 ± 10.28	Physiques (height); muscular strength (left hand grip, right hand grip); muscular endurance (sit-up per 60 s); muscular power (standing long jump); cardiorespiratory endurance (VO_2_max); flexibility (sit and reach); and agility (eyes-closed single-leg stance)
	Yoo et al., 2015 [35]	12	20.58 ± 0.79	11.08 ± 2.27	72.42 ± 9.96	Physiques (height, body fat, and muscle mass); muscular power (standing long jump)
	Yoo et al., 2011 [36]	8	21.87 ± 1.12	NR	78.03 ± 8.80	Physiques (height, body fat, and muscle mass); muscular power (standing long jump)
Female	Feng et al., 2020 [18]	7	20.00 ± 0.82	NR	62.67 ± 6.35	Physiques (height, BMI); cardiorespiratory endurance (VO_2_max); and isokinetic muscular strength (relative peak anaerobic power)
	Monks, 2016 [28]	16	20.00 ± 0.83	8.16 ± 1.91	64.15 ± 8.49	Physiques (height, body fat, and LBM); muscular endurance (sit-up per 60 s); muscular power (vertical jump); cardiorespiratory endurance (VO_2_max, HRmax, and all-out duration time); flexibility (sit and reach); and isokinetic muscular strength (average power, peak anaerobic power, peak drop, left knee joint flexion at 60°/s, left knee joint extension at 60°/s, right knee joint flexion at 60°/s, right knee joint extension at 60°/s, left knee joint flexion at 180°/s, left knee joint extension at 180°/s, right knee joint flexion at 180°/s, and right knee joint extension at 180°/s)
	Han, 2014 [37]	22	19.13 ± 1.29	6.81 ± 1.78	59.86 ± 3.27	Physiques (height, body fat, fat mass, and LBM); muscular endurance (sit-up per 60 s); muscular power (standing long jump); cardiorespiratory endurance (20-m MST); flexibility (sit and reach); isokinetic muscular strength (left knee joint flexion at 60°/s, left knee joint extension at 60°/s, right knee joint flexion at 60°/s, right knee joint extension at 60°/s, left knee joint flexion at 120°/s, left knee joint extension at 120°/s, right knee joint flexion at 120°/s, right knee joint extension at 120°/s, left knee joint flexion at 240°/s, left knee joint extension at 240°/s, right knee joint flexion at 240°/s, right knee joint extension at 240°/s, left hip joint flexion at 30°/s, left hip joint extension at 30°/s, right hip joint flexion at 30°/s, right hip joint extension at 30°/s, left hip joint flexion at 60°/s, left hip joint extension at 60°/s, right hip joint flexion at 60°/s, right hip joint extension at 60°/s, trunk joint flexion at 60°/s, trunk joint extension at 60°/s, trunk joint flexion at 120°/s, and trunk joint extension at 120°/s)
	Moon et al., 2016 [29]	10	19.50 ± 1.10	NR	61.95 ± 8.73	Physiques (height)
Female	Seo et al., 2015 [7]	12	18.90 ± 1.24	8.90 ± 2.23	59.80 ± 6.56	Physiques (height, body fat, fat mass, and LBM); muscular power (standing long jump); cardiorespiratory endurance (20-m MST); flexibility (sit and reach); and isokinetic muscular strength (relative peak anaerobic power, peak drop)
	Song et al., 2010 [33]	10	19.60 ± 1.30	NR	62.50 ± 7.20	Physiques (height, body fat, and fat mass); cardiorespiratory endurance (VO_2_max, HRmax); and isokinetic muscular strength (relative peak anaerobic power, peak drop, left knee joint flexion at 60°/s, left knee joint extension at 60°/s, right knee joint flexion at 60°/s, right knee joint extension at 60°/s, left knee joint flexion at 180°/s, left knee joint extension at 180°/s, right knee joint flexion at 180°/s, and right knee joint extension at 180°/s)
	Song et al., 2015 [38]	22	19.16 ± 1.33	NR	55.96 ± NR	Physiques (height, body fat, fat mass, LBM); muscular strength (hand grip); muscular endurance (sit-up per 60 s); muscular power (standing long jump); cardiorespiratory endurance (20-m MST); flexibility (sit and reach); and isokinetic muscular strength (left knee joint flexion at 60°/s, left knee joint extension at 60°/s, right knee joint flexion at 60°/s, right knee joint extension at 60°/s, left knee joint flexion at 120°/s, left knee joint extension at 120°/s, right knee joint flexion at 120°/s, right knee joint extension at 120°/s, left knee joint flexion at 240°/s, left knee joint extension at 240°/s, right knee joint flexion at 240°/s, right knee joint extension at 240°/s, trunk joint flexion at 60°/s, trunk joint extension at 60°/s, trunk joint flexion at 120°/s, trunk joint extension at 120°/s, trunk joint flexion at 240°/s, and trunk joint extension at 240°/s)

NR, not reported; MST, multistage shuttle-run test; BMI, body mass index; LBM, lean body mass; VO_2_max, maximal oxygen consumption per minute; and HRmax, maximal heart rate per minute.

**Table 3:** Pooled and estimated statistics for the Taekwondo athletes’ physical characteristics.

Sex	Variables	Total Sample Size	Pooled Mean ± SD	Estimated Error (%)
Male	Height	430	178.00 ± 5.27 (cm)	±0.28
	Weight	430	71.76 ± 11.84 (kg)	±1.56
	Percentage of body fat	236	12.67 ± 2.77 (%)	±2.79
	BMI	224	22.16 ± 1.51 (kg/m^2^)	±0.89
Female	Height	99	168.49 ± 5.48 (cm)	±0.64
	Weight	99	60.30 ± 5.88 (kg)	±1.92
	Percentage of body fat	82	23.25 ± 3.28 (%)	±3.05

SD: standard deviation; BMI: body mass index.

**Table 4:** Pooled and estimated statistics for the male Taekwondo athletes’ physical fitness.

Sex	Variables	Total Sample Size	Pooled Mean ± SD	Estimated Error (%)
Male	Hand-grip strength	53	44.68 ± 4.38 (kg)	±2.64
	Back strength	78	120.13 ± 19.59 (kg)	±3.62
	Sit-up per 30 s	62	30.52 ± 4.50 (times)	±3.67
	Sit-up per 60 s	141	57.41 ± 6.09 (times)	±1.75
	Sit and reach	203	15.95 ± 6.52 (cm)	±5.63
	Backward flexion	42	58.61 ± 6.87 (cm)	±3.54
	VO_2_max	129	52.71 ± 7.62 (mL/kg/min)	±2.49
	HRmax	67	179.95 ± 9.51 (bpm)	±1.26
	20-m MST	51	97.75 ± 6.63 (times)	±1.86
	Standing long jump	164	242.97 ± 13.90 (cm)	±0.88
	Vertical jump	117	54.54 ± 5.23 (cm)	±1.74
	Whole-body reaction time (light)	42	0.283 ± 0.027 (ms)	±2.89
	Whole-body reaction time (sound)	56	0.280 ± 0.032 (ms)	±3.01
	Eyes-closed single leg stance	65	35.70 ± 23.06 (s)	±15.71
	Anaerobic average power	73	534.70 ± 76.09 (watt)	±3.26
	Peak anaerobic power (relative value)	146	11.07 ± 1.31 (watt/kg)	±1.92
	Peak anaerobic power (absolute value)	73	720.08 ± 115.17 (watt)	±3.67
	Peak drop	119	48.71 ± 7.78 (%)	±2.87
	Isokinetic flexion muscle strength of the left knee joint (60°/s)	94	127.30 ± 23.66 (Nm)	±3.76
	Isokinetic extension muscle strength of the left knee joint (60°/s)	94	208.27 ± 33.26 (Nm)	±3.23
	Isokinetic flexion muscle strength of the right knee joint (60°/s)	94	131.13 ± 24.35 (Nm)	±3.75
	Isokinetic extension muscle strength of the right knee joint (60°/s)	94	214.13 ± 32.24 (Nm)	±3.04

SD, standard deviation; VO_2_max, maximal oxygen consumption per minute; HRmax, maximal heart rate per minute; and MST, multistage shuttle run test.

**Table 5:** Pooled and estimated statistics for the female Taekwondo athletes’ physical fitness.

Sex	Variables	Total Sample Size	Pooled Mean ± SD	Estimated Error (%)
Female	Sit-up per 60 s	60	54.20 ± 6.84 (times)	±3.19
	Sit and reach	72	21.33 ± 6.87 (cm)	±7.44
	VO_2_max	33	48.28 ± 5.68 (mL/km/min)	±4.01
	20-m MST	56	81.04 ± 12.32 (times)	±3.98
	Standing long jump	56	192.47 ± 14.25 (cm)	±1.94
	Peak anaerobic power (relative value)	45	9.22 ± 1.08 (watt/kg)	±3.43
	Peak drop	38	52.16 ± 6.14 (%)	±3.74
	Isokinetic flexion muscle strength of the left knee joint (60°/s)	70	97.77 ± 14.84 (Nm)	±3.56
	Isokinetic extension muscle strength of the left knee joint (60°/s)	70	172.82 ± 24.80 (Nm)	±3.36
	Isokinetic flexion muscle strength of the right knee joint (60°/s)	70	97.38 ± 15.93 (Nm)	±3.83
	Isokinetic extension muscle strength of the right knee joint (60°/s)	70	167.01 ± 25.70 (Nm)	±3.61
	Isokinetic flexion muscle endurance of the left knee joint (120°/s)	44	75.77 ± 6.95 (Nm/s)	±2.71
	Isokinetic extension muscle endurance of the left knee joint (120°/s)	44	125.80 ± 15.84 (Nm)	±3.72
	Isokinetic flexion muscle endurance of the right knee joint (120°/s)	44	73.62 ± 8.79 (Nm)	±3.53
	Isokinetic extension muscle endurance of the right knee joint (120°/s)	44	125.12 ± 15.65 (Nm)	±3.70
	Isokinetic flexion muscle power of the left knee joint (240°/s)	44	58.30 ± 7.41 (Nm)	±3.76
	Isokinetic extension muscle power of the left knee joint (240°/s)	44	90.90 ± 11.91 (Nm)	±3.87
	Isokinetic flexion muscle power of the right knee joint (240°/s)	44	56.36 ± 6.32 (Nm)	±3.31
	Isokinetic extension muscle power of the right knee joint (240°/s)	44	90.53 ± 11.42 (Nm)	±3.73
	Isokinetic flexion muscle strength of the trunk joint (60°/s)	44	143.45 ± 23.46 (Nm)	±4.83
	Isokinetic extension muscle strength of the trunk joint (60°/s)	44	154.62 ± 30.41 (Nm)	±5.81
	Isokinetic flexion muscle endurance of the trunk joint (120°/s)	44	143.08 ± 29.51 (Nm)	±6.09
	Isokinetic extension muscle endurance of the trunk joint (120°/s)	44	140.91 ± 27.78 (Nm)	±5.83

SD, standard deviation; VO_2_max, maximal oxygen consumption per minute; and MST, multistage shuttle-run test.

**Table 6:** Ninety-five percent confidence intervals for the Taekwondo athletes’ physical characteristics.

Sex	Variables	95% CI	1%	5%	10%	20%	30%	40%	50%	60%	70%	80%	90%	95%	99%
Male	Height (cm)	95% LV	165.2	168.8	170.7	173.1	174.7	176.2	177.5	178.8	180.3	181.9	184.3	186.2	189.8
		95% UV	166.2	169.8	171.7	174.1	175.7	177.2	178.5	179.8	181.3	182.9	185.3	187.2	190.8
	Weight (kg)	95% LV	43.1	51.2	55.5	60.7	64.4	67.6	70.6	73.6	76.9	80.6	85.8	90.1	98.2
		95% UV	45.3	53.4	57.7	62.9	66.7	69.9	72.9	75.9	79.1	82.8	88.1	92.4	100.4
	Percentage of body fat (%)	95% LV	18.8	16.9	15.9	14.7	13.8	13.0	12.3	11.6	10.9	10.0	8.8	7.8	5.9
		95% UV	19.5	17.6	16.6	15.4	14.5	13.7	13.0	12.3	11.6	10.7	9.5	8.5	6.6
	BMI (kg/m^2^)	95% LV	25.5	24.5	23.9	23.2	22.8	22.3	22.0	21.6	21.2	20.7	20.0	19.5	18.5
		95% UV	25.9	24.8	24.3	23.6	23.2	22.7	22.4	22.0	21.6	21.1	20.4	19.9	18.8
Female	Height (cm)	95% LV	154.7	158.4	160.4	162.8	164.5	166.0	167.4	168.8	170.3	172.0	174.4	176.4	180.2
		95% UV	156.8	160.6	162.5	165.0	166.7	168.2	169.6	171.0	172.4	174.2	176.6	178.6	182.3
	Weight (kg)	95% LV	45.5	49.5	51.6	54.2	56.1	57.7	59.1	60.6	62.2	64.1	66.7	68.8	72.8
		95% UV	47.8	51.8	53.9	56.5	58.4	60.0	61.5	63.0	64.5	66.4	69.0	71.1	75.2
	Percentage of body fat (%)	95% LV	30.2	27.9	26.7	25.3	24.3	23.4	22.5	21.7	20.8	19.8	18.3	17.1	14.9
		95% UV	31.6	29.4	28.2	26.7	25.7	24.8	24.0	23.1	22.2	21.2	19.8	18.6	16.3

BMI, body mass index; CI, confidence interval; LV, lower value; and UV, upper value.

**Table 7:** Ninety-five percent confidence intervals for the Taekwondo athletes’ physical fitness.

Sex	Variables	95% CI	1%	5%	10%	20%	30%	40%	50%	60%	70%	80%	90%	95%	99%
Male	Hand-grip strength (kg)	95% LV	33.3	36.3	37.9	39.8	41.2	42.4	43.5	44.6	45.8	47.2	49.1	50.7	53.7
		95% UV	35.7	38.7	40.3	42.2	43.6	44.8	45.9	47.0	48.2	49.5	51.5	53.1	56.0
	Back strength (kg)	95% LV	70.2	83.5	90.7	99.3	105.5	110.8	115.8	120.7	126.1	132.3	140.9	148.0	161.4
		95% UV	78.9	92.2	99.4	108.0	114.2	119.5	124.5	129.4	134.8	141.0	149.6	156.7	170.1
	Sit-up per 30 s (times)	95% LV	18.9	22.0	23.6	25.6	27.0	28.3	29.4	30.5	31.8	33.2	35.2	36.8	39.9
		95% UV	21.2	24.2	25.9	27.8	29.3	30.5	31.6	32.8	34.0	35.4	37.4	39.0	42.1
	Sit-up per 60 s (times)	95% LV	42.2	46.4	48.6	51.3	53.2	54.9	56.4	57.9	59.6	61.5	64.2	66.4	70.6
		95% UV	44.2	48.4	50.6	53.3	55.2	56.9	58.4	60.0	61.6	63.5	66.2	68.4	72.6
	Sit and reach (cm)	95% LV	−0.1	4.3	6.7	9.6	11.6	13.4	15.1	16.7	18.5	20.5	23.4	25.8	30.2
		95% UV	1.7	6.1	8.5	11.4	13.4	15.2	16.8	18.5	20.3	22.3	25.2	27.6	32.0
	Backward flexion (cm)	95% LV	40.6	45.2	47.7	50.8	52.9	54.8	56.5	58.3	60.1	62.3	65.3	67.8	72.5
		95% UV	44.7	49.4	51.9	54.9	57.1	59.0	60.7	62.4	64.3	66.5	69.5	72.0	76.7
	VO_2_max (mL/kg/min)	95% LV	33.7	38.9	41.6	45.0	47.4	49.5	51.4	53.3	55.4	57.8	61.2	63.9	69.1
		95% UV	36.3	41.5	44.3	47.6	50.0	52.1	54.0	56.0	58.0	60.4	63.8	66.6	71.8
	HRmax (bpm)	95% LV	155.6	162.0	165.5	169.7	172.7	175.3	177.7	180.1	182.7	185.7	189.9	193.3	199.8
		95% UV	160.1	166.6	170.0	174.2	177.2	179.8	182.2	184.6	187.2	190.2	194.4	197.9	204.3
	20-m MST (times)	95% LV	80.5	85.0	87.4	90.4	92.5	94.3	95.9	97.6	99.4	101.5	104.4	106.8	111.4
		95% UV	84.1	88.7	91.1	94.0	96.1	97.9	99.6	101.3	103.0	105.2	108.1	110.5	115.0
	Standing long jump (cm)	95% LV	208.5	218.0	223.0	229.1	233.6	237.3	240.8	244.4	248.1	252.5	258.7	263.7	273.2
		95% UV	212.8	222.2	227.3	233.4	237.8	241.6	245.1	248.6	252.4	256.8	262.9	268.0	277.4
	Vertical jump (cm)	95% LV	41.4	45.0	46.9	49.2	50.8	52.3	53.6	54.9	56.3	58.0	60.3	62.2	65.8
		95% UV	43.3	46.9	48.8	51.1	52.7	54.2	55.5	56.8	58.2	59.9	62.2	64.1	67.7
	Whole-body reaction time (light, ms)	95% LV	0.338	0.320	0.310	0.298	0.289	0.282	0.275	0.268	0.261	0.252	0.240	0.231	0.212
		95% UV	0.355	0.336	0.326	0.314	0.306	0.298	0.292	0.285	0.277	0.269	0.257	0.247	0.229
	Whole-body reaction time (sound, ms)	95% LV	0.346	0.324	0.313	0.299	0.288	0.280	0.272	0.263	0.255	0.245	0.230	0.219	0.197
		95% UV	0.363	0.341	0.330	0.315	0.305	0.297	0.288	0.280	0.272	0.261	0.247	0.236	0.214
	Eyes-closed single-leg stance (s)	95% LV	−23.6	−7.8	0.5	10.7	18.0	24.2	30.1	35.9	42.2	49.5	59.6	68.0	83.7
		95% UV	−12.3	3.4	11.7	21.9	29.2	35.5	41.3	47.1	53.4	60.7	70.9	79.2	95.0
Male	Anaerobic average power (watt)	95% LV	340.2	392.1	419.7	453.2	477.3	498.0	517.2	536.5	557.1	581.3	614.8	642.4	694.3
		95% UV	375.1	427.0	454.6	488.1	512.3	532.9	552.2	571.4	592.1	616.2	649.7	677.3	729.2
	Peak anaerobic power (relative value, watt/kg)	95% LV	7.8	8.7	9.2	9.8	10.2	10.5	10.9	11.2	11.5	12.0	12.5	13.0	13.9
		95% UV	8.2	9.1	9.6	10.2	10.6	11.0	11.3	11.6	12.0	12.4	13.0	13.4	14.3
	Peak anaerobic power (absolute value, watt)	95% LV	425.7	504.2	546.1	596.7	633.3	664.5	693.7	722.8	754.1	790.6	841.3	883.1	961.6
		95% UV	478.6	557.1	598.9	649.6	686.1	717.3	746.5	775.7	806.9	843.4	894.1	935.9	1014.4
	Peak drop (%)	95% LV	29.2	34.5	37.3	40.8	43.2	45.3	47.3	49.3	51.4	53.9	57.3	60.1	65.4
		95% UV	32.0	37.3	40.1	43.6	46.0	48.1	50.1	52.1	54.2	56.7	60.1	62.9	68.2
	Left knee joint flexion (60°/s, Nm) ^a^	95% LV	67.5	83.6	92.2	102.6	110.1	116.5	122.5	128.5	134.9	142.4	152.8	161.4	177.6
		95% UV	77.0	93.2	101.8	112.2	119.7	126.1	132.1	138.1	144.5	152.0	162.4	171.0	187.1
	Left knee joint extension (60°/s, Nm) ^a^	95% LV	124.2	146.8	158.9	173.5	184.1	193.1	201.5	210.0	219.0	229.5	244.2	256.3	278.9
		95% UV	137.6	160.3	172.4	187.0	197.5	206.6	215.0	223.4	232.4	243.0	257.6	269.7	292.4
	Right knee joint flexion (60°/s, Nm) ^a^	95% LV	69.5	86.1	95.0	105.7	113.4	120.0	126.2	132.4	139.0	146.7	157.4	166.3	182.9
		95% UV	79.4	96.0	104.8	115.6	123.3	129.9	136.1	142.2	148.8	156.6	167.3	176.1	192.7
	Right knee joint extension (60°/s, Nm) ^a^	95% LV	132.6	154.6	166.3	180.5	190.7	199.4	207.6	215.8	224.5	234.8	248.9	260.6	282.6
		95% UV	145.6	167.6	179.3	193.5	203.7	212.5	220.7	228.8	237.6	247.8	262.0	273.7	295.7
Female	Sit-up per 60 s (times)	95% LV	36.6	41.2	43.7	46.7	48.9	50.7	52.5	54.2	56.1	58.2	61.2	63.7	68.4
		95% UV	40.0	44.7	47.2	50.2	52.3	54.2	55.9	57.7	59.5	61.7	64.7	67.2	71.8
	Sit and reach (cm)	95% LV	3.8	8.4	10.9	14.0	16.1	18.0	19.7	21.5	23.3	25.5	28.5	31.0	35.7
		95% UV	6.9	11.6	14.1	17.1	19.3	21.2	22.9	24.7	26.5	28.7	31.7	34.2	38.9
	VO_2_max (mL/kg/min)	95% LV	33.1	37.0	39.1	41.6	43.4	44.9	46.3	47.8	49.3	51.1	53.6	55.7	59.5
		95% UV	37.0	40.9	42.9	45.4	47.2	48.8	50.2	51.7	53.2	55.0	57.5	59.6	63.4
	20-m MST (times)	95% LV	49.2	57.6	62.0	67.4	71.4	74.7	77.8	80.9	84.3	88.2	93.6	98.1	106.5
		95% UV	55.6	64.0	68.5	73.9	77.8	81.1	84.3	87.4	90.7	94.6	100.1	104.5	112.9
	Standing long jump (cm)	95% LV	155.6	165.3	170.5	176.7	181.3	185.1	188.7	192.3	196.2	200.7	207.0	212.2	221.9
		95% UV	163.1	172.8	177.9	184.2	188.7	192.6	196.2	199.8	203.7	208.2	214.5	219.6	229.4
	Peak anaerobic power (relative value, watt/kg)	95% LV	6.4	7.1	7.5	8.0	8.3	8.6	8.9	9.2	9.5	9.8	10.3	10.7	11.4
		95% UV	7.0	7.8	8.2	8.6	9.0	9.3	9.5	9.8	10.1	10.4	10.9	11.3	12.1
Female	Peak drop (%)	95% LV	35.9	40.1	42.3	45.0	47.0	48.7	50.2	51.8	53.4	55.4	58.1	60.3	64.5
		95% UV	39.8	44.0	46.2	48.9	50.9	52.6	54.1	55.7	57.3	59.3	62.0	64.2	68.4
	Left knee joint flexion (60°/s, Nm) ^a^	95% LV	59.8	69.9	75.3	81.8	86.5	90.5	94.3	98.1	102.1	106.8	113.3	118.7	128.8
		95% UV	66.7	76.8	82.2	88.8	93.5	97.5	101.2	105.0	109.0	113.7	120.3	125.7	135.8
	Left knee joint extension (60°/s, Nm) ^a^	95% LV	109.3	126.2	135.2	146.1	154.0	160.7	167.0	173.3	180.0	187.9	198.8	207.8	224.7
		95% UV	120.9	137.8	146.8	157.8	165.6	172.3	178.6	184.9	191.6	199.5	210.4	219.4	236.3
	Right knee joint flexion (60°/s, Nm) ^a^	95% LV	56.6	67.4	73.2	80.2	85.3	89.6	93.6	97.7	102.0	107.0	114.1	119.8	130.7
		95% UV	64.1	74.9	80.7	87.7	92.8	97.1	101.1	105.1	109.5	114.5	121.5	127.3	138.2
	Right knee joint extension (60°/s, Nm) ^a^	95% LV	101.2	118.7	128.0	139.4	147.5	154.5	161.0	167.5	174.5	182.6	193.9	203.3	220.8
		95% UV	113.2	130.8	140.1	151.4	159.6	166.5	173.0	179.5	186.5	194.7	206.0	215.3	232.8
	Left knee joint flexion (120°/s, Nm) ^a^	95% LV	57.5	62.3	64.8	67.9	70.1	72.0	73.7	75.5	77.4	79.6	82.6	85.1	89.9
		95% UV	61.7	66.4	68.9	72.0	74.2	76.1	77.8	79.6	81.5	83.7	86.7	89.3	94.0
	Left knee joint extension (120°/s, Nm) ^a^	95% LV	84.3	95.1	100.8	107.8	112.8	117.1	121.1	125.1	129.4	134.5	141.4	147.2	158.0
		95% UV	93.6	104.4	110.2	117.2	122.2	126.5	130.5	134.5	138.8	143.8	150.8	156.5	167.3
	Right knee joint flexion (120°/s, Nm) ^a^	95% LV	50.6	56.6	59.8	63.6	66.4	68.8	71.0	73.3	75.6	78.4	82.3	85.5	91.5
		95% UV	55.8	61.8	65.0	68.8	71.6	74.0	76.2	78.4	80.8	83.6	87.5	90.7	96.7
	Right knee joint extension (120°/s, Nm) ^a^	95% LV	84.1	94.8	100.4	107.3	112.3	116.5	120.5	124.5	128.7	133.7	140.5	146.2	156.9
		95% UV	93.3	104.0	109.7	116.6	121.5	125.8	129.7	133.7	137.9	142.9	149.8	155.5	166.1
	Left knee joint flexion (240°/s, Nm) ^a^	95% LV	38.9	43.9	46.6	49.9	52.2	54.2	56.1	58.0	60.0	62.4	65.6	68.3	73.4
		95% UV	43.3	48.3	51.0	54.3	56.6	58.6	60.5	62.4	64.4	66.7	70.0	72.7	77.7
	Left knee joint extension (240°/s, Nm) ^a^	95% LV	59.7	67.8	72.1	77.4	81.1	84.4	87.4	90.4	93.6	97.4	102.6	107.0	115.1
		95% UV	66.7	74.8	79.2	84.4	88.2	91.4	94.4	97.4	100.7	104.4	109.7	114.0	122.1
	Right knee joint flexion (240°/s, Nm) ^a^	95% LV	39.8	44.1	46.4	49.2	51.2	52.9	54.5	56.1	57.8	59.8	62.6	64.9	69.2
		95% UV	43.5	47.8	50.1	52.9	54.9	56.6	58.2	59.8	61.5	63.5	66.3	68.6	72.9
	Right knee joint extension (240°/s, Nm) ^a^	95% LV	60.6	68.4	72.5	77.5	81.2	84.3	87.2	90.0	93.1	96.8	101.8	105.9	113.7
		95% UV	67.3	75.1	79.3	84.3	87.9	91.0	93.9	96.8	99.9	103.5	108.5	112.7	120.5
Female	Trunk joint flexion (60°/s, Nm) ^a^	95% LV	81.9	97.9	106.4	116.8	124.2	130.6	136.5	142.5	148.8	156.3	166.6	175.1	191.1
		95% UV	95.8	111.8	120.3	130.6	138.1	144.4	150.4	156.3	162.7	170.1	180.4	189.0	205.0
	Trunk joint extension (60°/s, Nm) ^a^	95% LV	74.9	95.6	106.7	120.0	129.7	137.9	145.6	153.3	161.6	171.2	184.6	195.7	216.4
		95% UV	92.8	113.6	124.6	138.0	147.7	155.9	163.6	171.3	179.6	189.2	202.6	213.6	234.4
	Trunk joint flexion (120°/s, Nm) ^a^	95% LV	65.7	85.8	96.5	109.5	118.9	126.9	134.4	141.8	149.8	159.2	172.2	182.9	203.0
		95% UV	83.2	103.3	114.0	127.0	136.3	144.3	151.8	159.3	167.3	176.6	189.6	200.3	220.4
	Trunk joint extension (120°/s, Nm) ^a^	95% LV	68.1	87.0	97.1	109.3	118.1	125.7	132.7	139.7	147.3	156.1	168.3	178.4	197.3
		95% UV	84.5	103.4	113.5	125.7	134.6	142.1	149.1	156.2	163.7	172.5	184.7	194.8	213.7

VO_2_max, maximal oxygen consumption per minute; HRmax, maximal heart rate per minute; MST, multistage shuttle-run test; CI, confidence interval; LV, lower value; UV, upper value; ^a^, isokinetic muscular strength.

## Data Availability

No new data were created or analyzed in this study. Data sharing is not applicable to this study.
